# Natural Healing

**Published:** 1886-04

**Authors:** 


### HALL'S JOURNAL OF HEALTH

Vol. 33. APRIL, 1886. No. 4.

### NATURAL HEALING.

In the first number of the present volume of the *Journal*, some allusion was made to a class of persons who have acquired great celebrity as, what may be termed, natural healers.

Perhaps the most celebrated of these, at least in our day, was the late Doctor James R. Newton, whose marvelous cures attracted a world-wide attention. It was his custom during the more active period of hic professional life, to visit the great centres of population for ministrations to the afflicted. All were invited to avail themselves of the opportunity thus afforded, the rich and forehanded, for such commensurate reward as they could well afford, and the poor, "without money, and without price." I will relate a single case, which is avouched for by the well known Doctor J. V. Mansfield, and as nearly in his own words as I am able. Both gentlemen were sojourning in the city of Cincinnatti, and although known to each other as specialists of wide repute, they had never met. One afternoon Doctor M. called upon the great healer, stating that he could not overcome his desire to see and talk with him, and if possible be an eye witness to his method of cure. After an interchange of compliments and civilities of a most hearty and natural order, Doctor M. was invited to prolong his visit, with the expectation that some patient might drop in for treatment during his stay, and so indeed it happened.

The new comer was a man, past the middle age, led in by a little boy. Doctor Newton accosted him pleasantly, with "Well, sir, what can I do for you to-day." "Nothing, nothing, I reckon, Doctor, but my next neighbor, who is aware of my total blindness, has repeatedly urged me to come and see you, and says he has little pity for me because of my neglect of it, for he is foolish enough to believe you can restore my lost sight, but I have no faith in it myself. I thought I would come to you, if only to satisfy him of his mistake. Of course you can do nothing for me."

"How long is it since you lost your sight," enquired the Doctor, as he critically examined the patient's condition."

"It is now going on eighteen years.',

"Is this your little boy?"

"Yes, Doctor, it is."

"Then you have never seen him; would you like to see him to-day?"

"O Doctor, why do you ask me? Of all things in the world, what could equal that?"

"Well, then, in twenty minutes you shall see as plainly as you ever did."

With this the Doctor began to manipulate his patient in a manner familiar to such as have had occasion to observe a similar mode of treatment. In something less than twenty minutes the Doctor addressed him:

"Now open your eyes; what is that projection from the wall?"

"It seems—it *looks* like a mantel piece—yes it *is*; I *do* see it."

"What is that on the mantel?"

"It is a printed card."

"Read the large large line at the top."

The patient read it, "Is this a dream, or do I really see?" he asked himself, then turning his eyes to other objects on the wall, as if to "make assurance doubly sure," ventured at length to glance downward to his little boy, timidly, as if half in doubt of the reality of his restored vision; then with a quick, convulsive movement he seized and folded to his heart the astonished little guide, whose hour of liberation had come in the working of a miracle, which like all other recorded miracles, depended not the less upon the influence of natural laws, because those laws have been for the most part unappreciated and never thoroughly understood.

It is now some three years, speaking after the manner of the world, since the venerable Doctor Newton was laid at rest, but the equally venerable Doctor Mansfield is still doing his work amongst us, in a no less marvellous way. What is above recorded of Doctor Newton, is by no means an isolated instance of cure at his hands. There were very many instances within his eventful career as a natural healer, equally startling. If written out in detail they would fill volumes. Nor was he the only one of his time gifted in a like manner.

It is only a very short period since the world renowned "Swiss bone-setter" was a temporary resident of our sister city of Brooklyn, which she had been induced to visit, by a leading citizen who had availed himself of her remarkable powers for an afflicted member of his family. The cures reported of this lady, who, without any intimate knowledge of anatomical structure—without the use of instruments or medicines—reduced fractures and mended broken bones simply by "the laying on of hands," challenge belief in the minds of those who have had no experiences, in that direction. Yet they stand to-day as *truths*, questioned only by the ignorant or the bigoted, for there are *scientific*, no less than *religious* bigots.

A single instance of cure by this wonderfully gifted person, which the writer was mode acquainted with, will suffice as an illustration.

An accident had occasioned a deformity in the person of a young girl, at the hip, and the family physician placed her upon a stretcher, with a weight attached to one foot in the endeavor to lengthen the contracted limb, evenly with the other. The position was a rigid and a painful one, from which there was to be no near at hand relief.

After long suffering the Swiss bone-setter was called in, and throwing aside the instrument of torture, by simple manipulations, restored the injured part to its natuaal condition, without pain and with little loss of time. It may be asked, where is this divinely gifted person now? She has returned to her own country, and I am told her departure was hastened by the opposition of the *regulars* to a system of practice at outs with that which is prescribed by the *best surgical authority*, coupled with threats of prosecution for venturing to relieve the afflicted by unrecognized methods. "It was a famous victory."

In the next number of the *Journal*, it is my purpose to relate some well authenticated instances of cure by means of Clairvoyance, unless over ruled by the management. The readers of the *Jourual* ought to have the benefit of any well established fact of cure by whatever means, even though at variance with what, by courtesy, has been termed "medical science."

